# Assessment of Selected Anthropometric Parameters Influence on Balance Parameters in Children

**DOI:** 10.3390/medicina56040176

**Published:** 2020-04-14

**Authors:** Agnieszka Turon-Skrzypinska, Artur Uździcki, Tomasz Przybylski, Aleksandra Szylinska, Małgorzata Marchelek-Myśliwiec, Aleksandra Rył, Iwona Rotter

**Affiliations:** 1 Department of Medical Rehabilitation and Clinical Rehabilitation, Pomeranian Medical University, Żołnierska 54, 71-210 Szczecin; Poland; agi.skrzypinska@gmail.com (A.T.-S.); Aleksandra.szylinska@gmail.com (A.S.); ryl.ola@gmail.com (A.R.); iwrot@wp.pl (I.R.); 2 Department of Medical Rehabilitation and Clinical Rehabilitation, Student Scientific Society “KINESIS”, Pomeranian Medical University in Szczecin, Żołnierska 54, 71-210 Szczecin, Poland; tomek.przybylski94@gmail.com; 3 Department of Nephrology, Transplantology and Internal Medicine, Pomeranian Medical University, Al.Powstańców Wielkopolskich 72, 70-111 Szczecin, Poland; malgorzata.marchelek@gmail.com

**Keywords:** balance, stability, children, postural control

## Abstract

*Background and Objectives:* Balance is the ability of an organism to maintain its position in space. Balance disorders in children can lead to injuries and limited physical activity. Balance maintenance changes throughout puberty as well as in response to external factors. The study aimed to evaluate the influence of anthropometric parameters on balance in children aged 10 to 13 years. *Materials and Methods:* 308 children were accessed to eligibility to participate in the study. After considering the inclusion and exclusion criteria the study included 223 participants (123 boys and 100 girls) aged 10 to 13 from elementary schools in Szczecin. The stabilometry of examineted children was performed using the SIGMA balance platform. *Results:* It was shown that the balance parameters in children aged 10 to 13 worsen with increasing body mass and height, and do not correlate with age. *Conclusions:* Rapid diagnosis and identification of postural disorders in children make it possible to start targeted physical exercises and to make the therapeutic process more effective and complex. Future research is needed to obtain more data and draw conclusions crucial for physiotherapy practice.

## 1. Introduction

Balance is a broad term defined as the ability of the body to regain its default position in space after a destabilizing stimulus has stopped or as the ability to maintain its center of gravity over the area outlined by feet [1,2,3]. A similar term defined in terms of motion is the postural stability, i.e., the ability to maintain and control the center of gravity relative to the ground to prevent falls and enable effective execution of the intended motion in space. In elderly patients, imbalance is an important risk factor for falls, which can lead to permanent disability, especially with concomitant osteoporosis [4]. Balance evaluation is also important in children as even suboptimal balance parameters can increase the risk of falls, hamper psychomotor development and limit a child’s activity [5,6]. Balance development starts in infants, when the static postural reflexes and weak defense responses are already functioning, which are integrated at the level of the spinal cord and medulla oblongata. Around the age of 6 to 8 months, balance responses start to emerge, which later remain active throughout the lifetime. They are controlled by the cerebral cortex, subcortical nuclei, and cerebellum [7].

Three sensory systems are involved in maintaining balance, including proprioception, vision and vestibular system. Signals received by those systems must be detected and correctly analyzed; in physiological conditions, sensory deprivation from one or two different receptors (e.g., blindness or closing eyes) should not prevent a person from maintaining balance. Nevertheless, effectors are important as well—normal postural musculature provides the appropriate response to signals from receptors [5]. Optimal control and training of postural muscles significantly improve balance [8].

For objective evaluation of balance parameters, standardized diagnostic tests and measuring devices can be used, such as stabilometric platforms or contact force measuring mats [6,9]. The measured parameters such as pathway length of the center of pressure and deviation dynamics are often used to characterize postural balance in children [10].

It has been shown that balance training in young adults significantly improves the mechanisms responsible for balance maintenance [11]. Other factors may influence the development of balance; our study included children at school age when they are exposed to many factors negatively influencing posture and postural stability, such as poorly-sized school benches, distance to the blackboard, incorrect sitting, or carrying heavy backpacks [12]. Only a few studies are describing the evaluation of anthropometric parameters on postural stability in children. Identifying parameters affecting posture in children will enable future research on the most effective methods for diagnosis and management of balance disorders in children. It can also bring valuable insights into balance development in children, making it possible to diagnose any disturbances at the early stages.

This study aimed to evaluate the relationship between selected anthropometric parameters and balance in children aged 10 to 13 years.

## 2. Materials and Methods

**Scheme 1:**
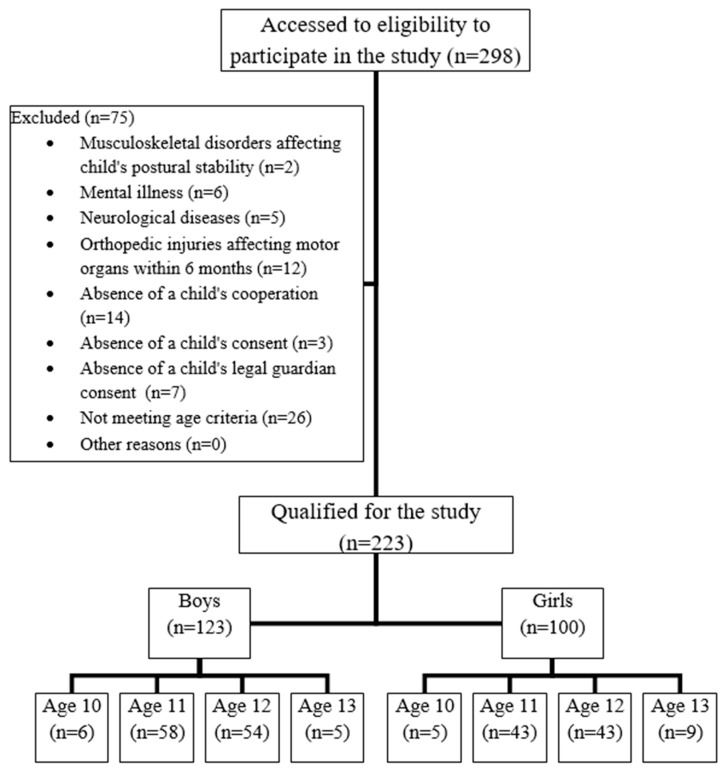
Selection of study participants.

308 children were accessed to eligibility to participate in the study. After considering the inclusion and exclusion criteria the study included 223 participants (123 boys and 100 girls) aged 10 to 13 (mean age = 11.51, SD = 0.68) from randomly chosen elementary schools in Szczecin (Scheme 1). The groups did not differ significantly with respect to age, body mass, and height.

The bioethics committee at the Pomeranian Medical University in Szczecin approved the research project No. KB-0012/14/15, on 02.02.2015. The study was conducted after obtaining written, informed consent by the children, school principals and parents. Both participants and their parents were informed about the aim, study outline and future use of the results. The participants were informed about their right to withdraw their consent to participate in the study without giving any reason. The physical examination was safe and non-invasive, i.e., without breaking the skin. The measurements were obtained according to ethical standards. The inclusion criteria included solely age 10–13, consent of a child and child’s legal guardian, child’s cooperation, absence of musculoskeletal disorders affecting postural stability and absence of mental illness, lack of neurological diseases or orthopedic injuries affecting motor organs within six months before the examination (Scheme 1). The measurements were conducted in the primary school of participating schools at a temperature of 22 °C. The children were wearing sports costumes (so that the clothes did not limit their movement), and the sex segregation was maintained. During the physical examination, the following were measured: body mass and height using medical scales with height rod SECA 220 and postural stability using the SIGMA balance platform. To acquire a precise measurement of a child’s height all children were instructed to stand at the scales barefoot, with heels, buttocks, and shoulders touching the scales, and with head in Frankfurt plane (the inferior margin of the left orbit and the upper margin of each ear canal or external auditory meatus were aligned parallelly to the ground level).

The stabilometry was performed using the SIGMA balance platform with a diameter of 42 cm and a weight of 6.5 kg. After each measurement, the platform was disinfected and stabilizing caps were applied (same caps each time). Then, the child was placed on the platform, paying attention to the correct position and distance between feet—feet were placed in equal distance from Y-axis marked on the platform, as indicated by the platform’s producer. Then the platform was calibrated using the producer’s software. Each measurement took about 30 s, and the child was asked to keep the dot displayed on the screen at the center. Using the software provided by the producer, all results were saved and stored anonymously. The statistical analysis was conducted using Microsoft Excel 2016 and Statistica 13.1. The descriptive statistics were obtained, and then correlations were calculated for the entire group as well as for each age group separately to eliminate the influence of the association between the age and the tested variables on the results. Stability parameters were checked using the Shapiro–Wilk test, which confirmed a normal distribution. The data were analyzed within groups using Pearson’s method. The statistical significance level was set for *p* ≤ 0.05.

## 3. Results

**Figure 1:**
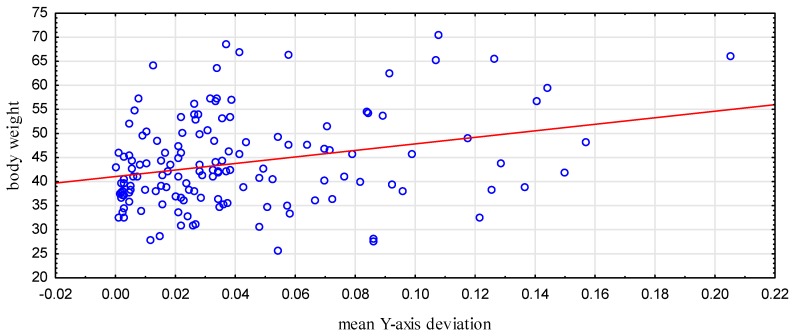
The relationship between body mass and the mean Y-axis deviation in 12-year-olds (*p* < 0.001).

**Figure 2:**
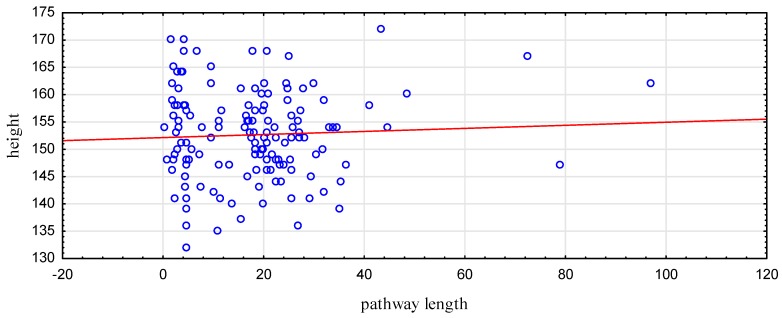
The relationship between the height and the pathway length in 12-year-olds (*p* = 0.047).

Correlations were conducted between anthropometric and stability parameters in boys and girls (Table 1 and Table 2). No correlations were found between age and balance parameters. For the entire study group, the statistically significant results were obtained only for 12-year-olds, probably because they were the most numerous age group. For the entire group of 12-year-olds, we found a statistically significant positive correlation with medium effect size between the body mass and the mean Y-axis deviation as well as a significant positive correlation with small effect size between the height and the pathway length (Figure 1 and Figure 2, respectively). However, when individual correlations were calculated for 12-year-old boys and girls separately (Table 3), only the body mass correlated positively with medium effect with balance parameters in a significant way (in each case with the mean Y-axis deviation, and for girls also in X-axis deviation). For almost all age groups, we found positive correlations between body mass and balance parameters using the Pearson’s method (coefficient as high as 0.75), indicating a large effect, but in most cases, the results were not statistically significant for *p* < 0.05. The strongest statistically significant correlation was the influence of the body mass on the Y-axis deviation in 12-year-old girls (Table 3). Additionally, in this group, the body mass correlated slightly with the mean X-axis deviation. No statistically significant correlation was found for the area.

In girls, the anthropometric parameters affected significantly more balance parameters as compared to boys (Table 3).

For the entire group, the height showed a significant correlation with balance as well. In both boys and girls, such relationships were observed, however, at this group size no statistical significance was shown (Table 2).

At ages 10 and 13 some small to medium-sized negative correlations were observed, but these were not significant (Table 2).

## 4. Discussion

Postural stability control and balance maintenance are essential for basic functioning [1] and hence their impaired development can hugely affect the child’s function later in life. As mentioned before, maintaining balance engages three systems, i.e., the vestibular system, vision, and proprioception. The received signals make it possible to adjust the spatial orientation and maintain balance.

In our study, we tried to evaluate the relationship between anthropometric parameters and postural stability in children aged 10 to 13. At this age, the child attends school and is exposed to many factors, which can negatively influence its posture and postural stability such as poorly-adjusted school bench size, distance to the blackboard, incorrect sitting position, carrying a heavy backpack, and hunching [13]. It is also believed that age 7 is critical for postural development [13]. Other authors, including Wolański et al. and Roncesvalles et al. consider postural stability to develop between the ages 8 to 9 [14,15]. There are also papers arguing that the locomotor and postural model in 7-year-olds is similar to adults [15,16,17]. Nevertheless, with new experiences and skills, it is possible to continue the development of all elements engaged in postural control, and keeping correct posture in children up to the age of 12 can be harder than in adults due to lacking integration of signals coming from different receptors [18,19]. In our study, we did not observe any correlation between age and anthropometric parameters. It might indicate that the center of gravity is stable as early as the age of 10 and does not change with age later on. That correlation, although not statistically significant, has been acknowledged in the cited literature.

One factor that influenced postural stability in a statistically significant way was the child’s body mass. In many studies, including ours, it has been established that large body mass in children negatively influences their balance and, as a consequence, leads to limited physical activity [20,21,22,23]. According to WHO age- and gender-specific growth reference charts, overweight in children and adolescents aged 5–19 years is defined as body mass index (BMI), +1 standard deviations (SD), and obesity as BMI +2 SD. [24] In our study the percentage of overweight boys and girls was 17.88% and 20%, respectively, and the percentage of obese boys and girls was 11.38% and 6%, respectively. The results obtained in Kułaga et al. say that the estimates of overweight prevalence in boys and girls aged 7–18 years in the Polish population, using the WHO definition, is 24.6% (23.66–25.52%) and 17.4% (16.58–18.15%), respectively, and obesity is seen in 8.8% (8.17–9.39%) of boys and 4.2% (3.84–4.68%) of girls [25]. In the study by Villarrasa-Sapiña et al., it was shown that obese children, compared to their peers with normal body mass, display longer pathways on the pressure platform test, which is reflected by poorer balance parameters [20].

Vision is considered an essential factor playing a key role in maintaining balance in adults [13]. However, many studies show that vision in children does not substantially affect stabilogram parameters. The study by Błaszczyk et al. shows that, in children aged 3 to 7, the vision has only a minor influence on the stability parameters, which proves that the vision does not play a considerable role in maintaining balance [1,13].

In the study by Peterson et al., it was shown that children up to the age of 12 cannot fully integrate signals coming from the eyes and the vestibula to keep postural stability [21]. Steindl et al. established that the full integration of visual and vestibular signals in maintaining upright posture does not develop until the age of 15 to 16 [22].

To sum up, coordination and cooperation with vision develop as late as up to the age of 16. In our study, we did not include stabilization platform tests with closed eyes, and thus we are unable to assess the impact of visual integration on postural stability; however, it should be kept in mind that visual disturbances can also affect balance parameters. In our study, visual disturbances constituted an exclusion criterium.

Another important issue in postural stability are posture problems, which are frequently encountered in school-aged children. When unnoticed or ignored, they can negatively affect balance development and stability in children, leading to poor balance and increased risk of falls and bone fractures in adult life [23]. It has been proven that patients with idiopathic scoliosis have poorer posture stability control compared to healthy individuals, and left-sided scoliosis leads to poorer stabilometric parameters in children [26,27]. In the study by Walicka-Cupryś et al., it has been established that increased posterior torso tilt and increased anterior pelvic tilt result in an abnormal load on the anterior parts of the feet, the left feet in particular, and poorer stabilometric parameters including sagittal-plane deviation [28]. In our study, we assumed increased lumbar lordosis and thoracic kyphosis to be the exclusion criteria, to eliminate the influence of those factors on our results.

Colne et al. compared prepubescent obese boys with normal-sized boys and showed an increased X-axis and Y-axis deviation in the former [29]. Body mass can also affect balance by causing abnormal posture [30]. This multidirectional influence has been confirmed in numerous studies evaluating the impact of the body mass on balance parameters [31,32]. In our study, we found a statistically significant correlation with the medium impact between the body mass and the mean Y-axis deviation in 12-year-olds, which confirmed the premise that obesity in children negatively affects their postural stability. Randomized clinical trials are being conducted investigating the effect of weight loss in obese children on their balance and neuromotor functioning, which will attempt to answer the question of whether the balance disturbances caused by obesity can resolve after returning to the normal body mass [33]. Additionally, our study showed a statistically significant influence of the height on the balance parameters.

As for today, there are no established norms for used balance parameters, as the method using a balance platform is still a novelty. Therefore, we could only calculate the correlations between the parameters—it could not be determined if the balance parameters are correct in each child. In future research, it may be useful to determine such norms in larger study populations, as it can make clinical decisions easier, providing additional information during children’s physiotherapy and training.

Our research shows that anthropometric parameters have an important influence on balance in children, which may suggest the need to broaden the research to obtain data on the impact of different parameters on balance, such as parietal height, shoulder width, pelvic girdle, width between interspinous fragments, and chest, shoulder, and forearm circumference.

## 5. Conclusions

Research on postural stability in children is crucial in both theory and clinical practice, especially for physiotherapists. Rapid diagnosis and identification of postural disorders in children make it possible to start targeted physical exercises and to make the therapeutic process more effective and complex. In each child, factors affecting the development and function of the balance system are different. It is possible to tailor exercise programs in individual cases of postural control dysfunction, taking into account the impact of factors such as the body mass, height, age, and sex on balance parameters. However, future research is needed to obtain more data and draw conclusions crucial for physiotherapy practice.

## Figures and Tables

**Table 1:** Comparison of anthropometric and stability parameters between boys and girls.

Variable		Boys (*n* = 123)				Girls (*n* = 100)			
		X	±SD	Min	Max	X	±SD	Min	Max
Age [y]		11.46	0.66	10	13	11.55	0.72	10	13
Body mass [kg]		41.97	11.08	25.1	70.8	42.14	8.8	24.5	77.0
Height [cm]		148.63	8.88	131	172	147.51	9.23	125	170
Mean deviation from the axis	X	0.072	0.06	0.004	0.308	0.07	0.06	0.001	0.29
	Y	0.05	0.06	0.0003	0.269	0.05	0.04	0.002	0.27
Pathway length		22.78	12.85	5.87	83.2	23.28	13.81	5.69	96.95
Area		0.88	0.56	0.1	2.76	0.89	0.49	0.1	2.49

X—mean; SD—standard deviation; Min—minimal value; Max—maximal value.

**Table 2:** Correlation between anthropometric and stability parameters for the entire study group.

Variable		Mean X-Axis Deviation	Mean Y-Axis Deviation	Pathway Length
Entire study group				
Age	R	−0.006	−0.05	0.07
	(p)	0.93	0.42	0.28
Children aged 10				
Body mass [kg]	R	−0.33	0.26	0.011
	(p)	0.37	0.72	0.97
Height [cm]	R	0.1	0.5	−0.27
	(p)	0.77	0.19	0.61
Children aged 11				
Body mass [kg]	R	0.18	0.03	0.12
	(p)	0.07	0.79	0.23
Height [cm]	R	0.16	0.02	0.07
	(p)	0.1	0.86	0.45
Children aged 12				
Body mass [kg]	R	0.17	0.37	0.13
	(p)	0.1	<0.001	0.2
Height [cm]	R	0.05	0.17	0.2
	(p)	0.6	0.09	0.047
Children aged 13				
Body mass [kg]	R	−0.03	0.46	−0.07
	(p)	0.92	0.17	0.84
Height [cm]	R	0.18	−0.29	−0.28
	(p)	0.59	0.37	0.4

R—correlation coefficient p—*p*-value.

**Table 3:** Correlations between anthropometric and stability parameters in boys and girls.

Variable			Boys				Girls		
			Mean X-axis Deviation	Mean Y-axis Deviation	Pathway Length	Area	Mean X-axis Deviation	Mean Y-axis Deviation	Pathway Length
Entire group	Age	R	0.03	0.01	0.13	0.07	−0.048	−0.1	0.01
		(p)	0.72	0.96	0.16	0.46	0.63	0.29	0.89
Age 10	Body mass [kg]	R	−0.1	0.53	0.47	0.46	−0.78	−0.67	−0.9
		(p)	0.83	0.34	0.39	0.4	0.25	0.31	0.19
	Height [cm]	R	0.25	0.75	0.41	0.47	−0.8	−0.63	−0.84
		(p)	0.64	0.19	0.45	0.39	0.24	0.34	0.22
Age 11	Body mass [kg]	R	0.2	0.04	0.2	0.17	0.17	−0.02	−0.06
		(p)	0.14	0.74	0.13	0.22	0.29	0.91	0.72
	Height [cm]	R	0.14	0.05	0.15	0.22	0.3	−0.11	−0.14
		(p)	0.3	0.66	0.27	0.1	0.053	0.49	0.38
Age 12	Body mass [kg]	R	0.03	0.33	0.1	0.13	0.37	0.45	0.17
		(p)	0.84	0.02	0.45	0.34	0.02	0.01	0.26
	Height [cm]	R	0.003	0.18	0.21	0.18	0.12	0.17	0.2
		(p)	0.98	0.2	0.13	0.2	0.43	0.26	0.2
Age 13	Body mass [kg]	R	−0.52	0.67	0.73	0.94	0.37	−0.26	−0.63
		(p)	0.41	0.31	0.28	0.18	0.44	0.59	0.2
	Height [cm]	R	−0.16	−0.02	−0.07	0.72	0.32	−0.75	−0.37
		(p)	0.79	0.97	0.9	0.27	0.5	0.14	0.44

R—correlation coefficient; p—*p*-value.
